# Microevolution of Burkholderia pseudomallei during an Acute Infection

**DOI:** 10.1128/JCM.01219-14

**Published:** 2014-09

**Authors:** Direk Limmathurotsakul, Matthew T. G. Holden, Paul Coupland, Erin P. Price, Narisara Chantratita, Vanaporn Wuthiekanun, Premjit Amornchai, Julian Parkhill, Sharon J. Peacock

**Affiliations:** a Department of Tropical Medicine and Hygiene, Faculty of Tropical Medicine, Mahidol University, Bangkok, Thailand; b Mahidol-Oxford Tropical Medicine Research Unit, Faculty of Tropical Medicine, Mahidol University, Bangkok, Thailand; c The Wellcome Trust Sanger Institute, Cambridge, United Kingdom; d Global and Tropical Health Division, Menzies School of Health Research, Darwin, Australia; e Department of Microbiology and Immunology, Faculty of Tropical Medicine, Mahidol University, Bangkok, Thailand; f Department of Medicine, Cambridge University, Addenbrooke's Hospital, Cambridge, United Kingdom

## Abstract

We used whole-genome sequencing to evaluate 69 independent colonies of Burkholderia pseudomallei isolated from seven body sites of a patient with acute disseminated melioidosis. Fourteen closely related genotypes were found, providing evidence for the rapid *in vivo* diversification of B. pseudomallei after inoculation and systemic spread.

## TEXT

Melioidosis is a common infection in South and East Asia and northern Australia that is caused by the soil-dwelling Gram-negative bacillus Burkholderia pseudomallei (1). The case fatality rate is high (range, 14% to 40%), despite the availability of effective antimicrobials, such as ceftazidime or a carbapenem drug (2, 3). In addition, patients who survive the initial stage of infection require 20 weeks of oral antibiotic therapy to prevent relapse (1). Relapse often affects the organ involved during the initial episode and is probably caused by a persistent nidus of infection after an apparent cure (4). B. pseudomallei may also become latent in the host following the initial inoculation event, and years or even decades may pass before the development of clinical manifestations (5). The mechanism(s) by which B. pseudomallei persists *in vivo* are unclear. We hypothesized that genetic diversification of B. pseudomallei within the host might provide insight into the mechanism(s) of persistence.

Whole-genome sequencing (WGS) has been used to evaluate within-host evolution of B. pseudomallei over protracted periods, including in a comparison of isolates from 4 patients with cultures performed at the time of primary infection and at relapse 6 months to 6 years later (6) and during chronic infection in a single patient over 12 years (7). Here, we focus on the short-term evolutionary events during the first 2 weeks of infection in a single individual. This was achieved by performing WGS of 69 colonies obtained from 7 different samples, the results of which were also compared with previously published multilocus variable-number tandem-repeat analysis (MLVA) data (8).

The study patient was a 22-year-old previously healthy male laborer who suffered a motorbike accident in northeast Thailand. He became febrile on day 3, and blood cultures taken on day 6 were positive for B. pseudomallei after 4 days of incubation. On day 11, multiple small pustules were noted on his forehead and both legs, consistent with disseminated melioidosis (9). He died on day 12. Ten individual B. pseudomallei colonies were picked from the primary culture plates from each of seven positive samples taken on day 11 (6 samples, from blood, respiratory secretions, urine, pus from pustule on right leg, left leg, and forehead) or on day 12 (1 sample, from wound swab from left thigh) (9). Primary colonies were obtained from the blood using an Isolator 10 lysis centrifugation tube (Oxoid, Basingstoke, Hampshire, United Kingdom). The laboratory isolate number was 3921, and the colonies were assigned the numbers C1 to C70 (see Table S1 in the supplemental material). This patient was referred to in a previous publication on B. pseudomallei MLVA as patient 19 (8) (see the detailed methodological description in the supplemental material).

Of the 70 B. pseudomallei colonies picked from primary culture plates of 7 clinical samples, one colony from the sample taken from a pustule on the right leg (C5) failed sequence quality checks and was excluded from further analysis. All of the colonies belonged to multilocus sequence type (MLST) 670 (ST670). The two isolates designated ST670 in the MLST database (http://bpseudomallei.mlst.net/) represent a colony from the left leg and blood of this patient (8). Comparative genomic analysis revealed that the C1 genome (the first B. pseudomallei colony isolated from a right leg wound swab) was similar in size and structure to previously sequenced B. pseudomallei isolates in the public sequence databases (10), containing two chromosomes of 4,044,293 bp and 3,117,372 bp that encoded 3,400 and 2,301 coding sequences (CDSs), respectively.

**FIG 1:**
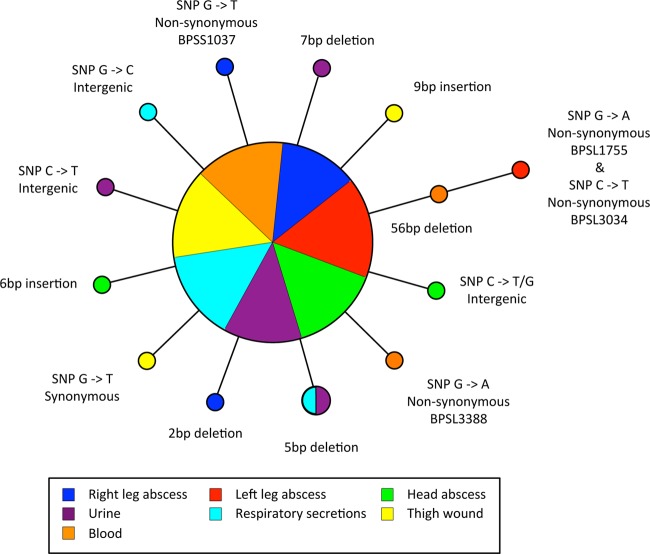
Parsimony phylogeny of melioidosis patient isolate genotypes: representation of the genotypes identified by whole-genome sequencing of multiple isolates obtained at different physiological sites. Isolates are color coded according to their clinical source. The sizes of the circles illustrate the relative sizes of the genotype populations (*n* = 55, *n* = 2, and *n* = 1). In total, 14 genotypes were observed, and the genetic events that distinguish each genotype from the founder genotype are indicated. The phylogeny is centered on the majority genotype inferred as the founder population.

Using the complete genome sequence of B. pseudomallei C1, we mapped the Illumina sequences and identified genetic variations in the serial patient isolates. The sequences of 55 colonies (80%) were identical to that of C1 and were considered to represent the putative founder genotype. The remainder were closely related variants of C1, falling into 13 separate WGS genotypes, which were distinguished by 14 different genetic events (6 small-scale indels [4 deletions and 2 insertions] and 8 single nucleotide polymorphisms [SNPs]) (Table 1 and Fig. 1). No large-scale insertions or deletions were observed in any of the genomes. Given the diversity of the population of B. pseudomallei in the environment and the lack of evidence for multiple ancestral genotypes, the possibility that the polymorphisms observed were from a coinfection of multiple strains with the same ST was very low (11). Aside from the putative founder genotype, each variant WGS genotype occurred once, with the exception of two pairs of colonies from different sites that contained the same event (Table 1). These pairs are likely to represent dissemination of the same strain to different organs rather than independent genetic changes that occurred in bacteria in different organs.

**TABLE 1:** SNPs and indels found in 14 B. pseudomallei isolates compared to the putative founder genotype (3921g-1)

Chromosome	Position	Nucleotide change	Colony no.	K96243^*a*^ orthologue	SNP or indel effect	CDS affected	Putative function
Large	196095	C→A	C56	BPSL0182	Synonymous substitution	Rod shape determining protein	Cell division
Large	730660	Deletion of GCACC	C35, C43	Intergenic			
Large	1894305	Deletion of CGACGAC	C34	Intergenic			
Large	2032551	G→A	C19	BPSL1755	Nonsynonymous substitution, Val132Met	Precorrin-4 C11-methyltransferase	Porphyrin metabolism
Large	2362053	Insertion of ACGGCG	C22	Intergenic			
Large	2485411	G→A	C39	BPSL2105	Synonymous substitution	PspA/IM30 family protein	Unknown
Large	3572138	C→T	C19	BPSL3034	Nonsynonymous substitution, Ala123Thr	Cell division protein MraZ	Cell division
Large	3990517	G→A	C69	BPSL3388	Nonsynonymous substitution, Ala268Val	Periplasmic amino acid binding transport protein	Nutrient acquisition
Large	4305318	G→C	C49	Intergenic			
Small	1406038	G→T	C8	BPSS1037	Nonsynonymous substitution, Leu427Met	Sugar transport protein	Nutrient acquisition
Small	1621217	Deletion of CTGGATGCTGGATGCTGGATGCTGGATGCTGGATGCTGGATGCTGGATGCTGGATG	C19, C70	Intergenic			
Small	2103217	C→T/G^*b*^	C24	Intergenic			
Small	2718281	Deletion of GC	C4	BPSS2034	Frameshift	Acetyl/propionyl-coenzyme A carboxylase alpha chain protein	Fatty acid biosynthesis
Small	3078443	Insertion of GAGTGCGGC	C55	BPSS2328	Duplication of tripeptide, His-Ser-Pro at position 1961	Multidomain beta keto-acyl synthase	Secondary metabolite production

a Burkholderia pseudomallei K96243 was a clinical isolate from northeast Thailand and was the first isolate with a complete genome (10).

b SNP heterogeneity, proportion G found as a minority variant at ∼20%.

Four of the eight SNPs (50%) resulted in nonsynonymous amino acid substitutions. Two of the CDSs affected were components of transport systems involved in nutrient acquisition, while the other two were involved in cell division and porphyrin metabolism. The potential biological effect of the other 4 SNPs were less obvious, as 2 were synonymous and 2 were located within intergenic regions. One of these intergenic SNPs on the small chromosome at position 2103217 was found to contain a mixed population of nucleotides (Table 1). The majority base detected was T (∼80% frequency; a C-to-T transition), but there was also evidence in the sequencing reads for this isolate of a second substitution of a G nucleotide (∼20% frequency) at this site. The heterogeneity at this site is intriguing, although the causes and effects of these two variants are unclear, as there are no gene or accompanying gene features, such as promoters, predicted in this region.

The six indels all represented length changes in short sequence repeat regions (see Table S1 in the supplemental material). Of these, four were within intergenic regions and two were within CDSs, both of which were predicted to alter translation of the encoded proteins. An isolate from the left thigh wound (C65) had a 9-bp insertion in the multidomain keto-acyl synthase CDS (BPSS2034), which resulted in an in-frame insertion of 3 amino acids (His-Ser-Pro). In contrast, an isolate from a right leg pustule (C4) had a 2-bp deletion in the acetyl/propionyl-coenzyme A carboxylase alpha chain protein CDS (BPSS2328) that resulted in a frameshift mutation, which potentially ablates protein expression.

Comparison of the WGS data with MLVA data published previously showed that these two techniques provided broadly similar levels of resolution to distinguish the B. pseudomallei population, but there was a lack of concordance between the two data sets (see Table S1 in the supplemental material). None of the indels detected using WGS were in variable-number tandem-repeat (VNTR) regions of the genome that were targeted by MLVA, and conversely, isolates that were predicted by MLVA to have variation within specific VNTRs were not identified from the WGS. It has been shown that the 933k, 2050k, and 3652k VNTR locus mutation rates range from ∼2 × 10^−4^ to 2 × 10^−6^ per generation (8), compared with SNP mutation rates of ∼10^−5^ to ∼10^−7^ per site per year measured in bacterial genome studies (12). It is therefore possible that some of the MLVA variations have occurred following culture passaging for DNA extraction. Three VNTR loci in the C1 genome (1764k, 20k, and 3152k) contained arrays that were larger than, or of a size similar to, the Illumina sequencing reads (100 bp); therefore, mapped reads did not bridge the VNTR sequence, and WGS could not be used to robustly check for variations at these loci. Our findings indicate that MLVA may be a useful adjunct to WGS for detecting additional fine-scale variations in closely related B. pseudomallei populations, where the short sequence read lengths of some WGS platforms may preclude variation prediction at loci containing large VNTR regions.

In conclusion, *in vivo* evolution of B. pseudomallei occurs within a short period during acute infection through SNPs and indel variations (which are particularly associated with tandem-repeat regions of the genome).

### Nucleotide sequence accession numbers.

Illumina sequence data for this project have been deposited in the European Nucleotide Archive under the study number ERP000173. The sequences and annotations of the two C1 chromosomes have been deposited in the EMBL database under accession numbers LK936442 and LK936443.

## Supplementary Material

Supplemental material
