# Supplementary material for: Microevolution of Burkholderia pseudomallei during an Acute Infection
Source: J Clin Microbiol. 2014 Sep;52(9):3418–21. doi: 10.1128/JCM.01219-14 (PMC4313173; doi:10.1128/JCM.01219-14)
Supplement: Supplemental material [file supp_52_9_3418__index.html]

Microevolution of Burkholderia pseudomallei during an Acute Infection — Supplemental material 

# Microevolution of Burkholderia pseudomallei during an Acute Infection

## Supplemental material

**Files in this Data Supplement:**

- Supplemental file 1 -

  Supplemental text

  PDF, 118K
- Supplemental file 2 -

  Table S1 (Summary of sequenced isolates and genomic variation)

  PDF, 94K
