# Supplementary material for: Microevolution of Burkholderia pseudomallei during an Acute Infection
Source: J Clin Microbiol. 2014 Sep;52(9):3418–21. doi: 10.1128/JCM.01219-14 (PMC4313173; doi:10.1128/JCM.01219-14)
Supplement: Supplemental material [file JCM.01219-14_zjm999093677so1.pdf]

Supplementary text for: Micro-evolution of *Burkholderia pseudomallei* during an acute infection, Limmathurotsakul et al.

Whole genome sequencing

DNA was extracted from *B. pseudomallei* isolates cultured in Trypticase soy broth for 24 hour at 37°C using the High Pure PCR template preparation kit (Roche Applied Science, Germany) according to the manufacturer's protocol. The first colony picked from the right leg wound swab (C1) was sequenced using the PacBio RS II instrument (Pacific Biosciences, Menlo Park, CA). All colonies were sequenced using the Illumina HiSeq2000 instrument (San Diego, CA) with 100 base paired-end reads and an amplification-free method (1). A high quality *de novo* reference genome sequence for C1 (isolate 3921g-1) was generated from the PacBio assembly refined with Illumina data (see below). Genome variation in the other colonies was identified by mapping the Illumina paired-end reads were against the C1 chromosomes (see below).

Generation of a high quality whole genome sequence for C1

One isolate, the first colony picked from the right leg wound swab (referred to as colony 1; C1), was picked for sequencing using the Pacific Biosciences RSII (PacBio) single molecule real time sequencing platform in order to generate a de novo reference sequence for variation analysis. PacBio continuous long reads were generated from SMRTbell libraries made with genomic DNA, sheared to approximately 18 kb with a MegaRuptor (Diagenode, Denville, NJ, USA) as per existing PacBio recommendation. The SMRTbell library was size-selected using BluePippin (Sage Science, Beverley, MA, USA) and bound to P4 polymerase. The bound complexes were loaded on to V3 SMRTcells using MagBeads, and sequenced using C2 chemistry and 180 min movies, again following current manufacturer's recommendation.

Primary filtering, performed on the RS Blade Center server, showed we had generated 800 Mb of data, consisting of 142,699 reads with a mean polymerase read length of 5.7 kb. Secondary analysis, using SMRTanalysis version v2.1.0, generated the following sub-read information; 152,434 sub-reads, mean sub-read length of 5.3 kb, N50 = 7.3 kb. De novo assembly for 1/1 was subsequently performed using the Hierarchical Genome Assembly Process (HGAP.2) that utilised the latest DAGCon based pre_assembler module. This generated an assembly consisting of two contigs that corresponded to two separate chromosomes, total genome size = 7.16 Mb. The estimated coverage of the draft assembly was 95.08 X, falling well within the current recommendation of 60 – 100 X worth of data for bacterial de novo assembly.

Errors in the PacBio de novo assembly were checked using Illumina data generated for this isolate (European Nucleotide Archive sample accession number ERS008778). The C1 Illumina data was mapped against the C1 PacBio de novo assembly as described in Croucher et al (2), and indels were identified using GATK (Genome Analysis Toolkit - www.broadinstitute.org/gatk). Where variation was called, the mapped reads displayed in the BAM file were inspected in Artemis to check for support for the assembly. No SNPs were detected, but 20 indel regions were identified; 17 were single base deletions and 3 were due to two base deletions. Manual inspection of the Illumina reads in these regions identified them as HGAP.2 generated mis-assemblies, and therefore the C1 sequence was corrected to produce a high quality whole genome sequence

Annotation from the *B. pseudomallei* K96243 reference strain (accession numbers BX571965 and BX571966) was compared with the C1 genome by BLASTN (3) and manually inspected and curated. Reciprocal best match using FASTA (4) was used to identify orthologous

protein coding sequences (CDSs). CDSs without BLAST matches in the K96243 genome were annotated using Prokka (Prokaryotic Genome Annotation System - www.vicbioinformatics.com/software.prokka).

In comparison to *B. pseudomallei* strain K96243, 95.5% of the CDSs on chromosome 1 and 96.8% of the CDSs on chromosome 2 had orthologous matches, with the majority of differences being found in novel Genomic Island (GI) regions. In total, 17 putative GI regions were identified in the C1 genome, 7 of which shared sequence similarity with GIs integrated at orthologous sites in the K96243 chromosomes. The chromosomes of C1 and K96243 were co-linear with the exception of one region on Chromosome 1 of C1, which contained a large inversion of ~0.9Mb relative to K96243 due to reciprocal recombination between CDSs encoding phage related proteins in GI regions.

Identification of variation

Illumina paired-end reads for all the other colonies were mapped against previously described high quality C1 chromosome sequences (accession numbers LK936442 and LK936443) using SMALT as described in Croucher et al. (2), and indels were identified using GATK. Where variation was called, the appropriate BAM file was inspected in Artemis to check for support and confirm the prediction. In the case to the heterogeneous base identified in C24 at position 2,103,217 of chromosome 2, this was confirmed from the BAM file, and the percentage nucleotide frequency of mixture estimated from inspection of the reads. No evidence of mis-mapping was found at this site that would account for the heterogeneity. The genetically similar pairs of colonies from different sites were unlikely to result from contamination of samples. Precautions to prevent contamination were taken in all study laboratories.

For the comparison of the MLVA results previously described (5) with the WGS data, the VNTR loci in the C1 genome were identified from the primer sequences (6). Six loci were demonstrated by Price *et al.* to be variant in our collection: 1764k, 20k, 2050k, 3152k, 3652k and 933k. Inspection of these loci in the C1 genome revealed that they were all composed of perfect tandem repeat arrays, and that they ranged in size from 63 bp to 120 bp. Manual inspection in Artemis of the mapped reads across the VNTR was carried out to identify possible MLVA predicted variants. Three of the C1 loci, 1764k, 20k and 3152k, contained arrays larger, or of a similar size to the Illumina sequencing reads (100 bp); therefore mapped reads did not bridge the VNTR sequence and could not be used to robustly check for variation at these loci. The 3 remaining loci, 2050k, 3652k and 933k, contained tandem repeat arrays of 63 bp, 72 bp and 72 bp respectively, which were sufficiently small to align across the VNTR and predict the MLVA genotype. Therefore, we were able to align reads across the VNTRs and flanking DNA and reliably check for variation. In total, 8 isolates with MLVA predicted variation at these 3 loci were checked. In only 2 isolates in right leg pustule (C3) and head pustule (C27), 2050k+1 mutation was supported by the WGS data. The failure of the in silico methods employed to detect these MLVA repeats reflects the inherent technical difficulties of mapping short sequencing reads to complex repetitive sequences.

Illumina sequence data for this project has been deposited in the European Nucleotide Archive under the study number ERP000173. The sequences and annotation of the two C1 chromosomes have been deposited in the EMBL database under accession numbers LK936442 and LK936443.

References

1. **Kozarewa I, Ning Z, Quail MA, Sanders MJ, Berriman M, Turner DJ.** 2009. Amplification-free Illumina sequencing-library preparation facilitates improved mapping and assembly of (G+C)-biased genomes. Nature Methods. 6:291-5.
2. **Croucher NJ, Harris SR, Fraser C, et al.** 2011. Rapid pneumococcal evolution in response to clinical interventions. Science. 331:430-4.
3. **Holden MT, Titball RW, Peacock SJ, et al.** 2004. Genomic plasticity of the causative agent of melioidosis, *Burkholderia pseudomallei*. Proc Natl Acad Sci U S A. 101:14240-5.
4. **Altschul SF, Gish W, Miller W, Myers EW, Lipman DJ.** 1990. Basic Local Alignment Search Tool. J Mol Biol. 215:403-10.
5. **Price EP, Hornstra HM, Limmathurotsakul D, et al.** 2010. Within-host evolution of *Burkholderia pseudomallei* in four cases of acute melioidosis. PLoS Pathog. 6:e1000725.
6. **U'Ren JM, Schupp JM, Pearson T, et al.** 2007. Tandem repeat regions within the *Burkholderia pseudomallei* genome and their application for high resolution genotyping. BMC Microbiol. 30:23
