# Supplementary material for: Microevolution of Burkholderia pseudomallei during an Acute Infection
Source: J Clin Microbiol. 2014 Sep;52(9):3418–21. doi: 10.1128/JCM.01219-14 (PMC4313173; doi:10.1128/JCM.01219-14)
Supplement: Supplemental material [file JCM.01219-14_zjm999093677so2.pdf]

Supplementary Table. Summary of the sequenced isolates and the genomic variation

Colony	ENA Sample Accession	Specimen	Date of isolation	VNTR variant	Variation predicted by WGS in comparison to 3921g-1 (C1)
C1	ERS008778	Rt leg W/S	17 July 2006	3152k †	-
C2	ERS008779	Rt leg W/S	17 July 2006	3652k *	-
C3	ERS008780	Rt leg W/S	17 July 2006	2050k	-
C4	ERS008781	Rt leg W/S	17 July 2006	1764k †	Chromosome 2, position 2718281, 2bp deletion of GC
C6	ERS008783	Rt leg W/S	17 July 2006	-	-
C7	ERS008784	Rt leg W/S	17 July 2006	-	-
C8	ERS008785	Rt leg W/S	17 July 2006	-	Chromosome 2, position 1406038, G -> T
C9	ERS008786	Rt leg W/S	17 July 2006	3652k *	-
C10	ERS008787	Rt leg W/S	17 July 2006	-	-
C11	ERS008788	Lt leg W/S	17 July 2006	-	-
C12	ERS008789	Lt leg W/S	17 July 2006	-	-
C13	ERS008790	Lt leg W/S	17 July 2006	-	-
C14	ERS008791	Lt leg W/S	17 July 2006	-	-
C15	ERS008792	Lt leg W/S	17 July 2006	20k †	-
C16	ERS008793	Lt leg W/S	17 July 2006	-	-
C17	ERS008794	Lt leg W/S	17 July 2006	-	-
C18	ERS008795	Lt leg W/S	17 July 2006	-	-
C19	ERS008796	Lt leg W/S	17 July 2006	2050k *	Chromosome 1, positions 2032551, G -> A; 3572138, C -> T. Chromosome 2, 1621217, 56 bp deletion of CTGGATGCTGGATGCTGGATGCTGGATGCTGGATGCTGGATGCTGGATGCTGGATG
C20	ERS008797	Lt leg W/S	17 July 2006	-	-
C21	ERS008798	Head W/S	17 July 2006	-	-
C22	ERS008799	Head W/S	17 July 2006	-	-
C23	ERS008800	Head W/S	17 July 2006	3652k *	-
C24	ERS008801	Head W/S	17 July 2006	-	Chromosome 2, position 2103217, C -> T/G (heterozgous SNP G ~20% varient)
C25	ERS008802	Head W/S	17 July 2006	-	-
C26	ERS008803	Head W/S	17 July 2006	-	-
C27	ERS008804	Head W/S	17 July 2006	2050k	-

C28	ERS008805	Head W/S	17 July 2006	-	-
C29	ERS008806	Head W/S	17 July 2006	1764k †	-
C30	ERS008807	Head W/S	17 July 2006	-	-
C31	ERS008808	Urine	17 July 2006	-	-
C32	ERS008809	Urine	17 July 2006	-	-
C33	ERS008810	Urine	17 July 2006	-	-
C34	ERS008811	Urine	17 July 2006	-	Chromosome 1, position 1894305, 7 bp deletion of CGACGAC
C35	ERS008812	Urine	17 July 2006	1764k †	Chromosome 1, position 730660, 5bp deletion GCACC
C36	ERS008813	Urine	17 July 2006	-	-
C37	ERS008814	Urine	17 July 2006	-	-
C38	ERS008815	Urine	17 July 2006	-	-
C39	ERS008816	Urine	17 July 2006	-	Chromosome 1, position 2485411, C -> T
C40	ERS008817	Urine	17 July 2006	933k *	-
C41	ERS008818	Tracheal suction	17 July 2006	-	-
C42	ERS008819	Tracheal suction	17 July 2006	-	-
C43	ERS008820	Tracheal suction	17 July 2006	1764k †	Chromosome 1, position 730660, 5bp deletion GCACC
C44	ERS008821	Tracheal suction	17 July 2006	-	-
C45	ERS008822	Tracheal suction	17 July 2006	2050k *	-
C46	ERS008823	Tracheal suction	17 July 2006	-	-
C47	ERS008824	Tracheal suction	17 July 2006	-	-
C48	ERS008825	Tracheal suction	17 July 2006	-	-
C49	ERS008826	Tracheal suction	17 July 2006	-	Chromosome 1, position 4305318, G -> C
C50	ERS008827	Tracheal suction	17 July 2006	-	-
C51	ERS008828	Thigh W/S	18 July 2006	-	-
C52	ERS008829	Thigh W/S	18 July 2006	-	-
C53	ERS008830	Thigh W/S	18 July 2006	-	-
C54	ERS008831	Thigh W/S	18 July 2006	-	-
C55	ERS008832	Thigh W/S	18 July 2006	-	Chromosome 2, position 3078443, 9bp insertion of GAGTGCGGC
C56	ERS008833	Thigh W/S	18 July 2006	-	Chromosome 1, position 196095 C -> A
C57	ERS008834	Thigh W/S	18 July 2006	-	-
C58	ERS008835	Thigh W/S	18 July 2006	1764k †	-
C59	ERS008836	Thigh W/S	18 July 2006	-	-
C60	ERS008837	Thigh W/S	18 July 2006	-	-
C61	ERS008838	Isolator	17 July 2006	-	-
C62	ERS008839	Isolator	17 July 2006	-	-

C63	ERS008840	Isolator	17 July 2006	-	-
C64	ERS008841	Isolator	17 July 2006	-	-
C65	ERS008842	Isolator	17 July 2006	-	-
C66	ERS008843	Isolator	17 July 2006	-	-
C67	ERS008844	Isolator	17 July 2006	-	-
C68	ERS008845	Isolator	17 July 2006	-	-
C69	ERS008846	Isolator	17 July 2006	20k †	Chromosome 1, position 3990517, G -> A
C70	ERS008847	Isolator	17 July 2006	-	Chromosome 2, 1621217, 56 bp deletion of CTGGATGCTGGATGCTGGATGCTGGATGCTGGATGCTGGATGCT GGATGCTGGATG

* Not supported by the sequence data

† Unable to examine due to the size of the VNTR
